# A Novel Study on the Self-Assembly Behavior of Poly(lactic-*co*-glycolic acid) Polymer Probed by Curcumin Fluorescence

**DOI:** 10.1021/acsomega.1c06919

**Published:** 2022-03-08

**Authors:** Hanine Zakaria, Riham El Kurdi, Digambara Patra

**Affiliations:** Department of Chemistry, American University of Beirut, Beirut 1107-2020, Lebanon

## Abstract

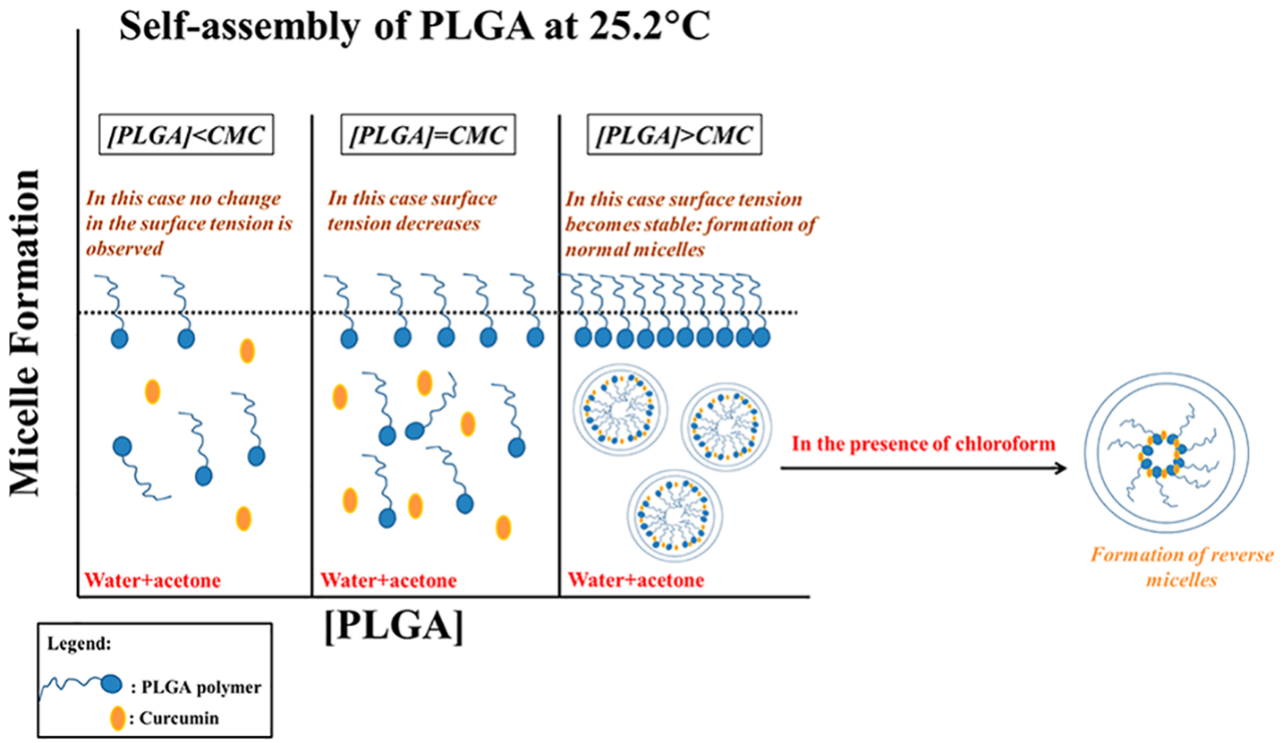

Understanding the self-assembly behavior of block copolymers is of great importance due to their usefulness in a wide range of applications. In this work, the physical properties of poly(lactic-*co*-glycolic acid) (PLGA polymer) are studied for the first time in solution using the fluorescence technique and curcumin as a molecular probe. First, curcumin at a concentration of 2 μM was added to different concentrations of PLGA, and the fluorescence of curcumin was tracked. It was found that the critical micellar concentration (CMC) was equal to 0.31 g/L and the critical micellar temperature (CMT) was obtained to be 25 °C. Furthermore, an insight on the effect of NaCl salt on the CMC value of PLGA is assessed through curcumin probing. A decrease in the CMC has been observed with the increase in the concentration of NaCl, which could be due to the salting out effect. Moreover, in order to understand the aggregation behavior of PLGA in different solutions, CMC experiments were investigated using chloroform as a solvent. Results showed that the solvent does not affect the CMC value of the polymer; however, it only affects the shape of the obtained micelle forming a reversed micelle. Finally, fluorescence quenching of curcumin with hydrophobic cetyl-pyridinium bromide (CPB) and hydrophilic KI quenchers was established, where it was proved that curcumin is located near the hydrophobic pocket of the Stern layer of the PLGA micelle.

## 1 Introduction

Smart polymers that possess responsive properties have gained a great interest in the last few decades because of their critical role in nanoscience and drug delivery applications.^1^ Poly(lactic-*co*-glycolic acid) (PLGA) is a synthetic copolymer of poly(lactic acid) (PLA) and poly(glycolic acid) (PGA).^2^ PGA is considered more hydrophilic compared to PLA due to the absence of additional asymmetrical methyl groups.^3^ Due to their usage in a wide range of applications in chemistry, pharmacy, and medicine, scientist paid a great attention on understanding the self-assembly aspects of block copolymers.^1^

Polymeric micelles are the result of the self-assemblage of amphiphilic polymers. These micelles have a unique set of polymers characteristics such as outstanding biocompatibility, low toxicity, and the solubility enhancement ability of poorly water-soluble drugs.^4^

In fact, micillization can occur above a certain concentration of polymer known as the critical micellar concentration and above a certain temperature known as the critical micellar temperature.^5,6^ When in an aqueous solution, the associate hydrophobic group and the hydrophilic group are left exposed to the solvent, and the structure is known in this case as a “normal” micelle.^7^ However, in a nonpolar solvent, the hydrophilic group is poorly solvated. This results in the formation of the interior of the aggregate, and the hydrophobic group surrounds therefore the formed polar core which is responsible for the solubility of the aggregate. Thereby, the formed structure is denoted as a “reverse micelles”.^7^ Indeed, in nonpolar solvents, the CMC value is not well-defined as in the aqueous medium because the aggregation number of reverse micelles is small, which make its determination difficult.^7^

In this manner, different methods were established in the literature for the aim of studying the CMC and CMT changes in block copolymers. These methods comprise the Fourier transform infrared (FTIR) spectroscopy technique,^8^ surface tension measurements,^9^ the DPH solubilization method,^10^ surface plasmon resonance,^11^ and fluorescence probing.^12^ In the latter method, pyrene molecules were always used and have proved to be a powerful tool as a fluorescence probe. In fact, pyrene is lethal to the kidneys and liver. It is also known that the pyrene molecule affects numerous existing functions in fish and algae. Thus, it was the need to find a fluorescence molecule having less toxic effects.^13^ For this purpose, curcumin is being developed to be used as a fluorescence probe for the determination of the polymer’s physical properties. Curcumin is a bioactive polyphenol derived from the rhizome of the *Curcuma longa*—a turmeric plant.^14^ Curcumin-based fluorescent probes can overcome the inadequacies of organic fluorescent dyes, such as low quantum yield, poor lipophilicity, and poor photostability, and also avoid possible interference of other substances with similar structures because of its high sensitivity and molecular targeting ability.^15^ In fact, curcumin had proven its efficiency as fluorescence probe in the estimation of CMC and CMT values for block copolymers such as poly(ethylene oxide)-*block*-poly(propylene oxide)-*block*-poly(ethylene oxide)^1^ and chitosan oligosaccharide lactate;^16^ thus, it serves as an excellent fluorescence probe for studying the self-assembly process. PLGA is emerging as a preferred polymer for drug delivery applications. However, there is not much literature detailing how this polymer aggregates and/or self-assembles in an aqueous environment, which will be crucial to understanding the drug–PLGA interaction and delivery mechanism. In this study, this has been further expanded upon; and curcumin was utilized as an external fluorescence probe in order to understand the self-assembly behavior of PLGA. To the best of our knowledge, until now the physical properties of PLGA have not been thoroughly studied or published.

## 2 Materials and Methods

### 2.1 Materials

Poly(lactic-*co*-glycolic acid (PLGA), curcumin, and sodium chloride (NaCl) were obtained from Sigma-Aldrich and used as received. The used solvents (chloroform, methanol, and acetone) were of HPLC grade and obtained from Sigma-Aldrich.

### 2.2 Sample Preparation

#### 2.2.1 CMC Sample Preparation

A stock solution of PLGA (1.8 mg/mL) was prepared in an acetone–water mixture. Likewise, a stock solution of 1 mM curcumin (*m* = 1.105 mg) was dissolved in methanol. Subsequently, dilutions were made as desired. The CMC study was investigated by substituting the acetone–water mixture with chloroform, and the effect of sodium chloride salt was also established.

For this purpose, fluorescence measurements for 10 samples of different PLGA concentrations in the range of 0–0.54 g/L were conducted. Curcumin’s concentration was maintained constant at 2 μM in all the samples.

To study the effect of salt on the CMC of PLGA, sodium chloride’s concentration was increased from 10 to 50 and then 150 mM.

To prove the effectiveness of curcumin as a fluorescence probe, the CMC study was conducted using pyrene as a fluorescence probe instead of curcumin.

#### 2.2.2 CMT Sample Preparation

For the CMT study, one sample was prepared where the PLGA and curcumin concentrations were maintained fixed at 0.4 8g/L and 2 μM, respectively. Fluorescence measurements for this sample were done by varying the temperature from 10 to 80 °C with 5 °C increments.

#### 2.2.3 Quenching Study

For the quenching experiment, PLGA and curcumin concentrations were kept constant at 0.48 g/L and 2 μM, respectively.

As for using KI as the quencher, the concentrations used were 0, 0.2, 0.4, 0.6, and 1 M. As for cetyl-pyridinium bromide (CPB), the concentrations used were as follows: 0, 50, 100, 200, 500, 800, and 1000 μM.

### 2.3 Instrumentation

Steady state fluorescence measurements were conducted by a Jobin–Yvon–Horiba fluorimeter. Emission and excitation slits were both set at 5 nm, and the excitation wavelength was set at 425 nm. All the fluorescence measurements were recorded over a wavelength range of 440–700 nm. The fluorometer was equipped with a 100 W xenon lamp and an R-928 detector working at 950 V. For temperature regulation, a thermostat was coupled to the fluorometer sample holder. An external thermometer was used for measuring the temperature. The width of the used cuvette was 1 cm.

## 3 Results and Discussion

The CMC and CMT experiments, in addition to the quenching study, were established by measuring the emission intensity of curcumin at λ_ex_ = 425 nm in the emission range 440–700 nm.

### 3.1 Self-Assembly and Critical Micelle Concentration

Even though micellization is a spontaneous process, it only begins above a certain concentration of the polymer known as the critical micellar concentration (CMC), at a fixed temperature. To determine this concentration, several physical properties such as surface tension, electrical conductivity, or osmotic pressure can be tracked as a function of polymer concentration.^1^ To examine the CMC of PLGA, curcumin was used as a fluorescence probe to study the aggregation behavior of PLGA.

The emission intensity of curcumin in the presence of different PLGA concentrations was measured at room temperature, excited at λ_ex_ = 425 nm in the emission range of 440–700 nm. It is commendable to note that the fluorescence emission intensity for a sample containing only PLGA polymer was measured and found to be negligible. It was found that the error is within 10%, meaning that the fluorescence is only due to curcumin’s presence.

**Figure 1:**
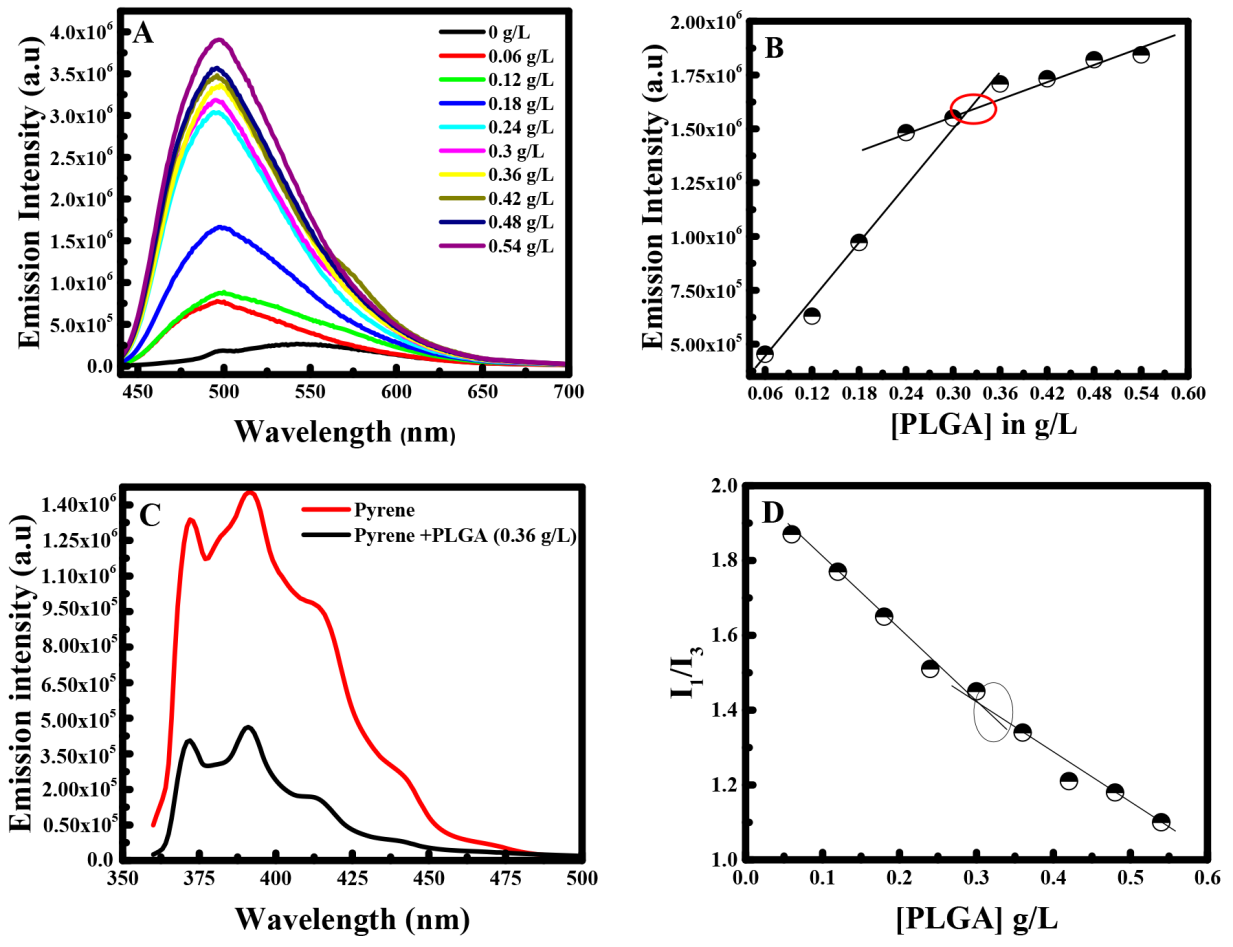
(A) Fluorescence emission spectra of curcumin at different concentrations of PLGA. (B) Fluorescence emission intensity of curcumin at λ_ex_ = 425 nm versus concentration of PLGA. (C) Fluorescence emission spectra of pyrene in the absence and the presence of PLGA (0.36 g/L). (D) *I*_1_/*I*_3_ of pyrene at λ_ex_ = 330 nm versus concentration of PLGA.

As shown in Figure 1A, the emission spectra of curcumin showed a peak at ∼498 nm after excitation at 425 nm. A blue shift from ∼545 nm (in the absence of polymer) to ∼496 nm was observed at higher concentrations of PLGA. The change in the fluorescence intensity at 498 nm with increase in PLGA concentration is shown in Figure 1B. It is obvious that the emission intensity of curcumin increases in two different ways. In the beginning, the emission intensity increases rapidly to a certain concentration, where it continued to increase, thus with smaller slope. Such a break in the fluorescence intensity vs the PLGA concentration can be attributed to the aggregation/micellization of PLGA. Therefore, it resembles the CMC value, which is estimated to be ∼0.31 ± 0.01 g/L. The observed blue shift and the increase in the fluorescence intensity indicate that curcumin experiences a more nonpolar environment in the formed PLGA micelles. This signifies the incorporation of curcumin into the hydrophobic core of PLGA micelles.

To prove the efficiency of curcumin as a fluorescence probe in the determination of the CMC value, the same experiment was carried out using pyrene as a fluorescence probe. For this purpose, the emission intensity of pyrene (*C* = 2 μM) in the absence of PLGA and the presence of 0.36 g/L PLGA was measured. As shown in Figure 1C, the addition of PLGA altered only the emission intensity of pyrene without any variation in the wavelength. Henceforward, the CMC of PLGA was established by varying the concentration of PLGA in the presence of 2 μM pyrene. The ratio of *I*_1_/*I*_3_ vs the concentration of PLGA is depicted in Figure 1D. Hence, when using pyrene the CMC value was equal to 0.31 ± 0.01 g/L. This value is equal to the CMC value obtained using curcumin. These results prove the efficiency of curcumin as a fluorescent probe in the determination of the polymer’s physical properties. Hence, the establishment of the CMC value will help to define the minimum amount of surfactant essential to reduce the maximum surface tension of the solvent, in addition to quantifying the needed concentration for drug delivery studies in further experiments.

### 3.2 Self-Assembly and Critical Micelle Temperature

**Figure 2:**
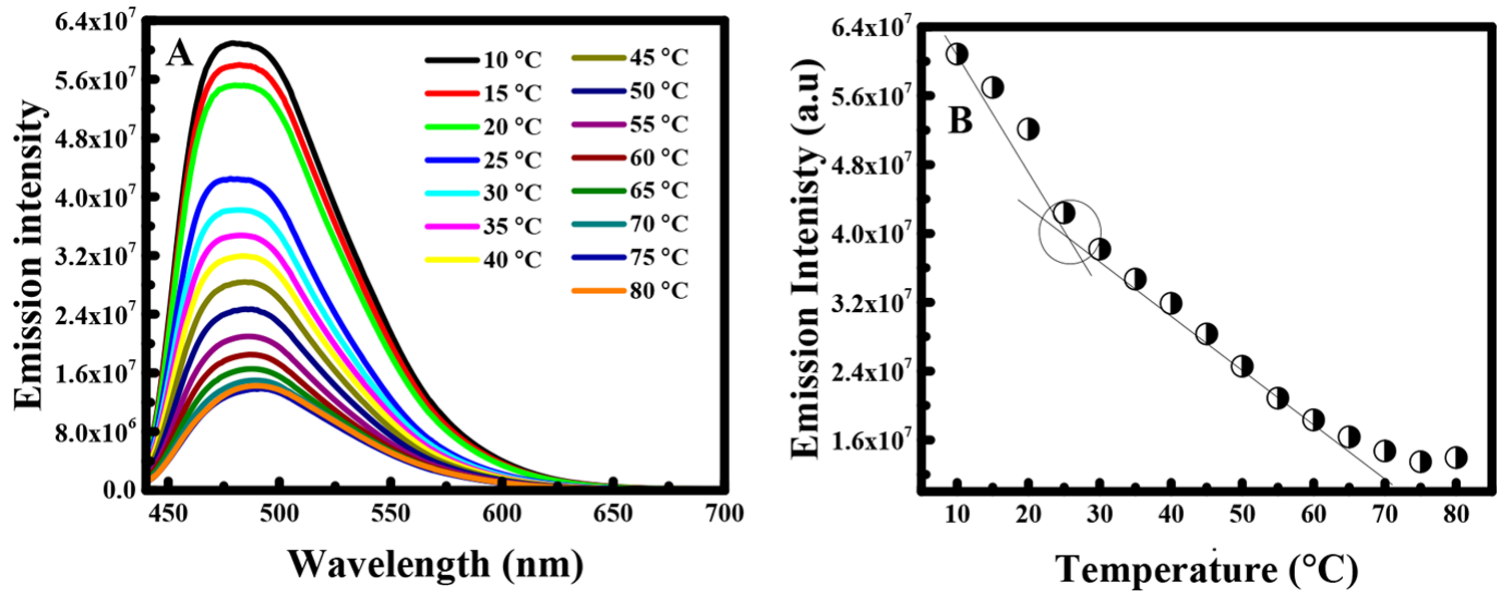
(A) Fluorescence spectra of PLGA solution *C* = 0.48 g/L at various temperatures in the range of 10–80 °C. (B) Plot of fluorescence emission intensity vs the temperature.

Due to the strong dependence of the CMC on temperature, the concept of the CMT has been extensively used.^17^ Temperature induces a crucial effect on the micellization process; thus, we decided to evaluate the CMT of PLGA at a concentration (0.48 g/L) that exceeded the CMC value of PLGA (0.31 g/L) obtained in the above-mentioned CMC experiment. The representative fluorescence spectra of the PLGA solution at a concentration of 0.48 g/L were recorded at different temperatures over the range 10–80 °C. As shown in Figure 2A, the emission intensity decreases with the increase of the temperature. The maximum of the emission intensity at 482 nm vs the temperature is depicted in Figure 2B.

In fact, at low temperature two essential aspects were present. On the one hand, PLGA did not associate in aqueous solution, and on the other hand, curcumin was not solubilized in a hydrophobic environment. Therefore, the fluorescence intensity was strong. Thus, at high temperature, the formation of micelles is encouraged, inducing the solubilization of curcumin in the hydrophobic micelle interior. Hence, the entrapment of curcumin in the hydrophobic core of the PLGA polymer diminishes the emission intensity of curcumin. Therefore, we can say that there is a distinct temperature at which fluorescence intensity declines dramatically, indicating the aggregation of PLGA into micelles. This break point can therefore be allocated as CMT. In this case, it was obtained at 25.2 °C.

### 3.3 Effect of NaCl Salt on the CMC of PLGA

**Figure 3:**
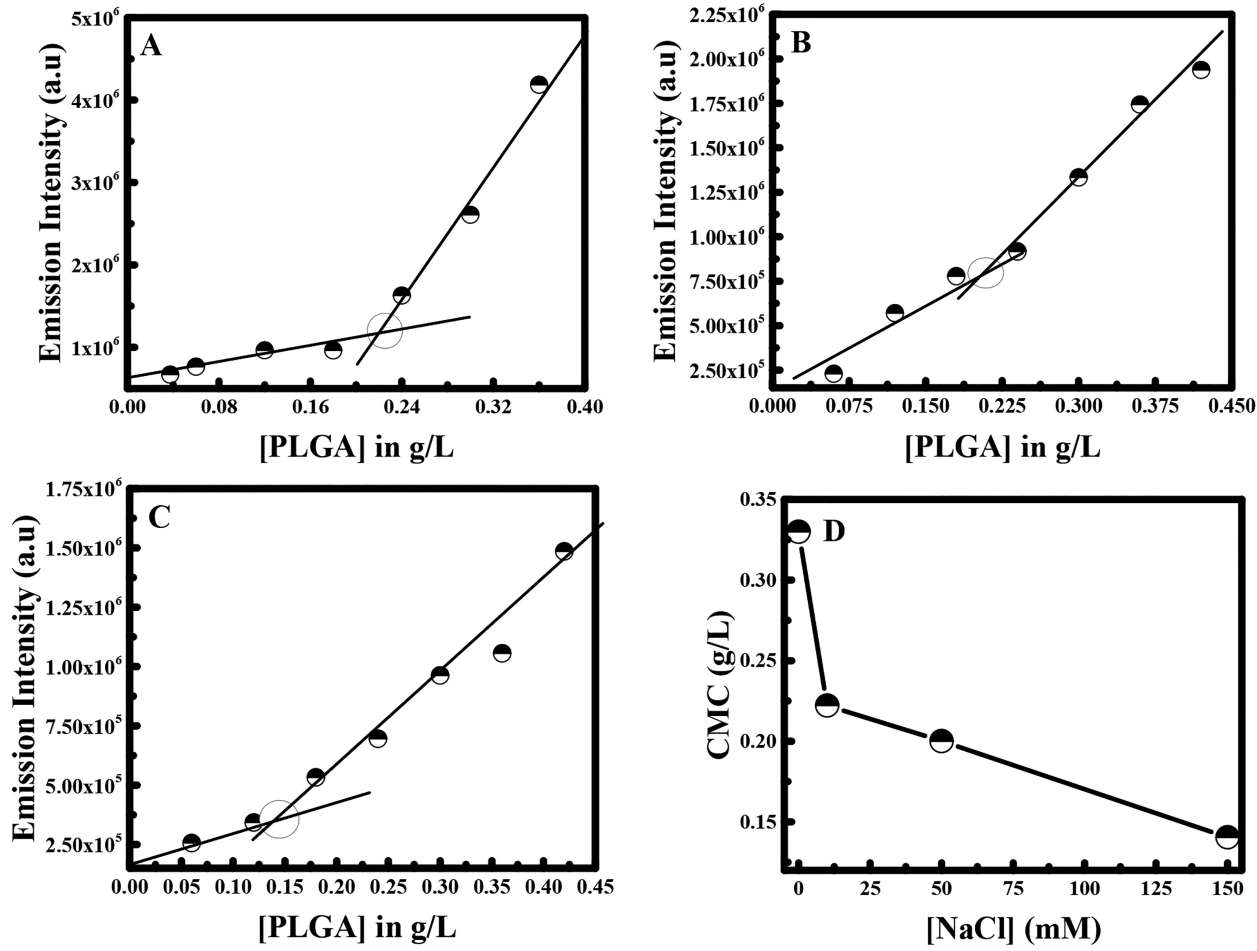
Fluorescence emission intensity of curcumin at λ_ex_ = 425 nm plotted versus concentration of PLGA in the presence of (A) 10 mM; (B) 50 mM; and (C) 150 mM NaCl. (D) Change in the CMC value with increased concentration of NaCl.

To study the effect of the ionic strength on the interaction of curcumin with PLGA, different concentrations of NaCl were used. NaCl was used since it is the commonly used salt and found in physiological conditions; therefore, it may influence the self-assembly of PLGA when it is used for drug delivery application. The examined concentrations of NaCl were 10, 50, and 150 mM. The fluorescence intensity of curcumin was monitored at 498 nm for each NaCl concentration in the presence of different PLGA concentrations as shown in Figure 3A–C. In the absence of NaCl, the CMC value was equal to 0.31 g/L. Hence, the CMC value was lowered by around twofold from 0.31 to 0.14 g/L as the NaCl concentration reaches 150 mM (see Figure 3D).

Certainly, this change in the CMC value was expected, as it is widely found in earlier comparable studies that NaCl drops the CMC value of the polymer. Hence, a study conducted by Desai et al. showed similar results when using pluronic polymer. In fact, the presence of sodium chloride had boosted the hydrophobicity in the PPO moiety and thereby lowered the hydrophilicity of the PEO moiety, leading to the formation of micelles at low concentration.^9^ Consequently, the reduction in the CMC value in the presence of NaCl salt induces the enhancement of micelles formation. Hence, in our case NaCl is pushing out the PLGA polymer from the aqueous phase, thus improving the micelle formation. This latter effect of NaCl salt is known as salting-out effect.^1^

### 3.4 Solvent Effect on CMC Values

To study the effect of the solvent on the micellization of the polymer, this experiment was repeated while dissolving PLGA in an organic nonpolar solvent, which is chloroform.

To understand the interaction of PLGA with curcumin in the presence of chloroform, various concentrations of PLGA were prepared in the range of 0–0.54 g/L, where curcumin’s concentration remained constant at 2 μM.

**Figure 4:**
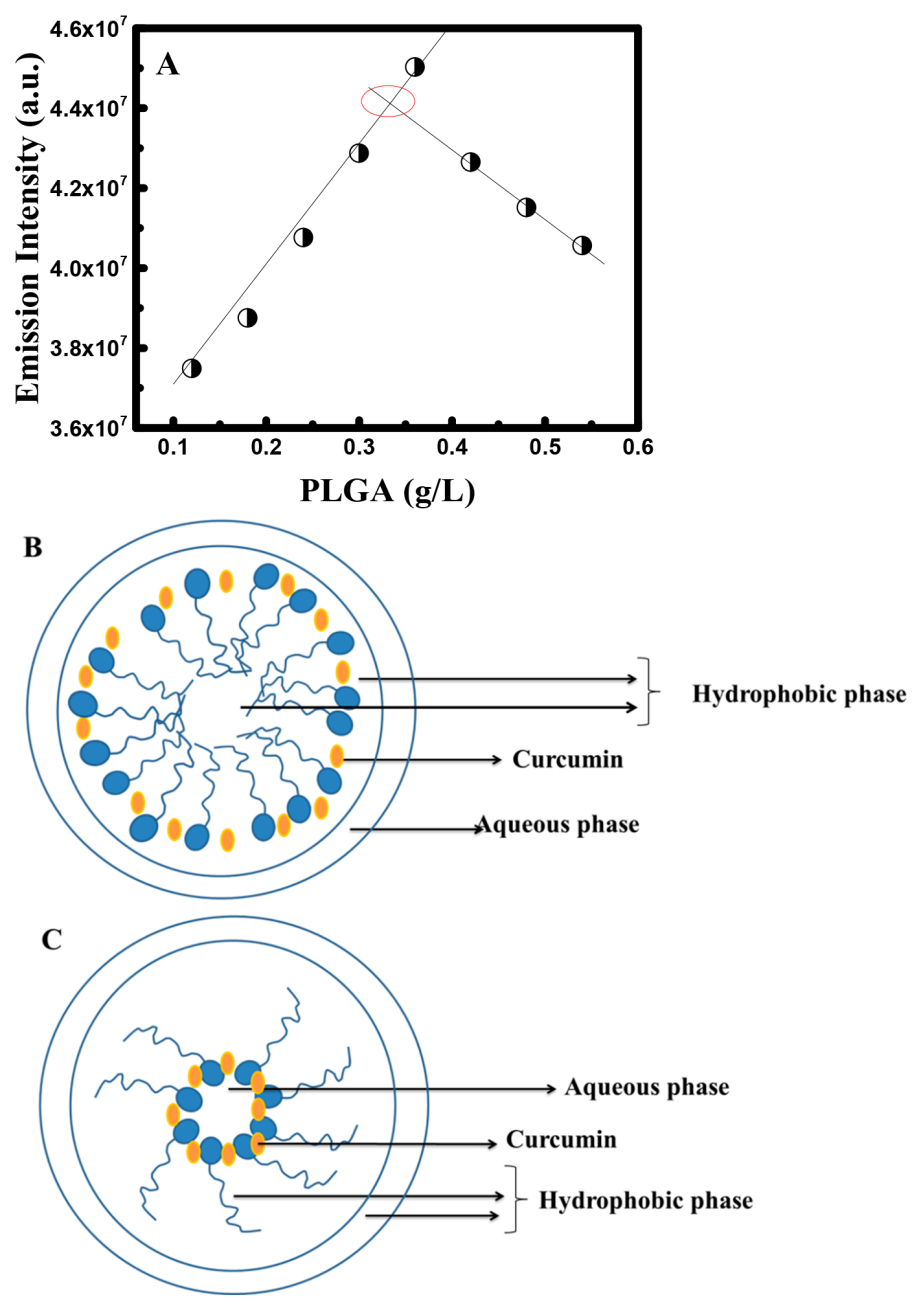
(A) Fluorescence emission intensity of curcumin at different concentrations of PLGA in the presence of chloroform. (B) normal structure of the micelle and (C) reverse micelle structure.

As shown in Figure 4A, the emission intensity of curcumin increased proportionally within the increase in the PLGA concentration until it reaches a maximum at 0.33 g/L, and then it starts to decrease gradually. This maximum concentration is related to the CMC value that was equal to 0.33 g/L. The CMC value found in the presence of chloroform was almost equal to the CMC value obtained when dissolving the PLGA in a water–acetone mixture. Thus, the main difference was in the change in the emission intensity, where a decrease in emission intensity was observed above the CMC value when using chloroform.

Indeed, as PLGA concentration increases, a greater number of PLGA molecules start coming together and bind to curcumin in solution. Such an assembly process helps the hydrophobic long chain group to associate with curcumin and improves its fluorescence. And so, the fluorescence intensity continued to increase with the increase of the polymer concentration until 0.33 g/L and started to decrease for *C* > 0.33 g/L.

This change in the emission intensity reveals that the assembly of PLGA ultimately creates aggregation or a structure similar to a reversed micelle. However, here we talk about an aggregated form where the solvent enhances the formation of a reversed micelle, meaning that the outside environment is highly hydrophobic, and the core is hydrophilic or less nonpolar (see Figure 4B,C).

In fact, due to the presence of phenolic and enolic groups in the curcumin molecule, curcumin prefers an environment of a regular micelle (hydrophobic core). Hence, the increase in the hydrophilicity of the micelle core diminishes the fluorescence. This is due to the fact that the fluorescence of curcumin is decreased in a polar medium compared to in a nonpolar medium. Nonetheless, this kind of interaction is not observed in an aqueous environment when water is used as a solvent for PLGA.

### 3.5 Quenching Study

To gain an insight about the accessibility of curcumin into the PLGA micelle, fluorescence quenching experiments were conducted.

For this purpose, KI and CPB quenchers were used. In fact, CPB is a hydrophobic quencher; its ion (cetrylpyridinium ion (CPy^+^)) is an electron acceptor, which quenches the fluorescence of probe molecules by the electron transfer mechanism.^18^ Thus, KI is a hydrophilic quencher, due to its negative I^–^ that prefers to stay in the aqueous phase.^1,19^

Hence, the fluorescence intensity of curcumin was measured at different concentrations of both quenchers. The fluorescence quenching of curcumin was measured using the Stern–Volmer relationship under steady state conditions.^20^

**Figure 5:**
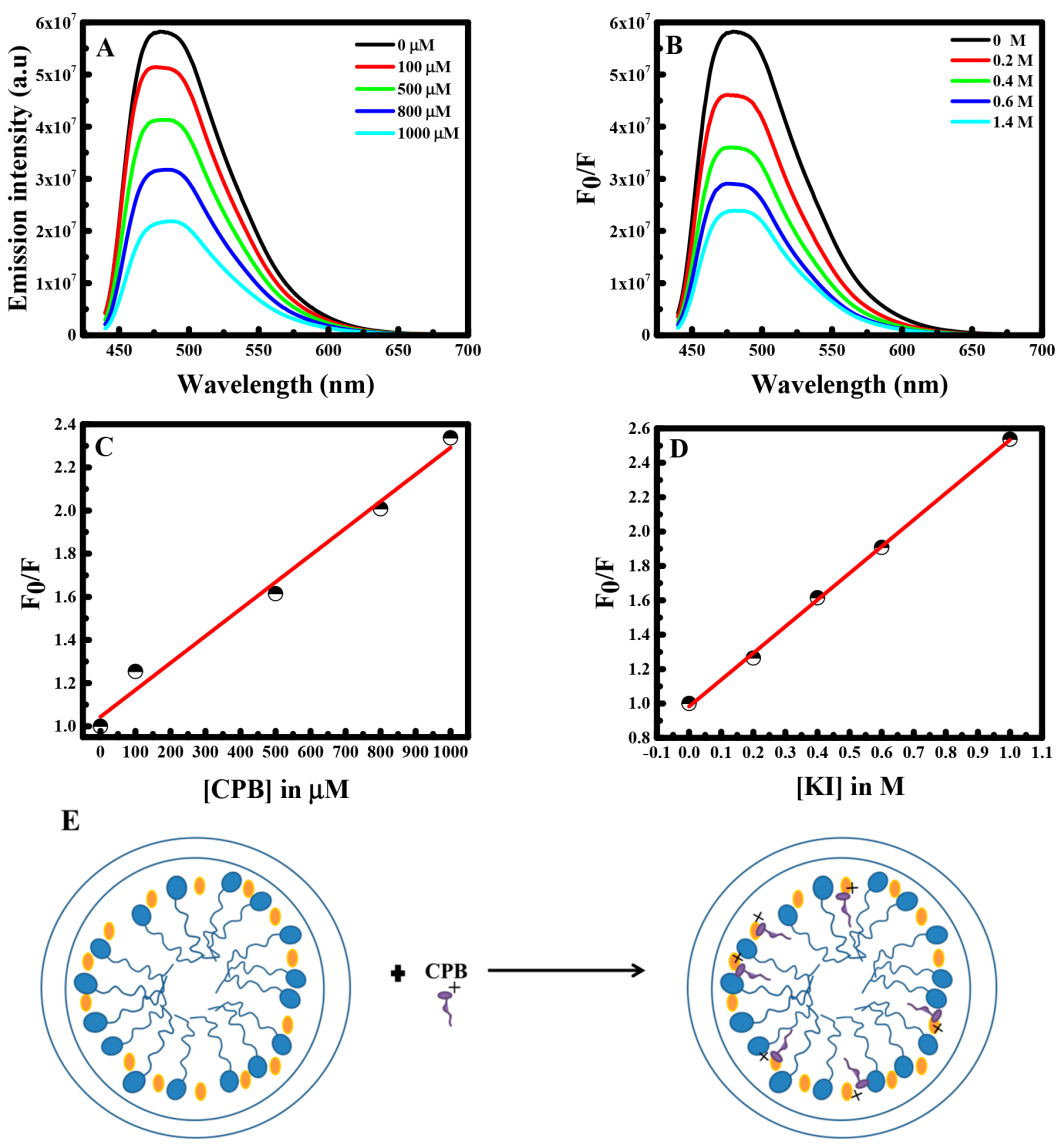
Fluorescence emission spectra of curcumin in the PLGA polymer at (A) various CPB concentrations and (B) various KI concentrations. (C) Stern–Volmer plot at various concentrations of CPB. (D) Stern–Volmer plot at various concentrations of KI. (E) Schematic representation of curcumin quenching in the presence of CPB.

As shown, in Figure 5A,B the fluorescence intensity of curcumin decreases as the concentration of KI and CPB quenchers increases, and the maximuim emission wavelength was almost unaltered. Actually, when using CPB, curcumin acts as an electron donor, where an electron in the excited state is transferred from its aromatic ring to an electron deficient N^+^ atom of CPB.^20^ Therefore, the CPB tail intercalates into the hydrophobic part of the PLGA micelles and remains at the Stern layer with its charged moiety exposed at the surface. Thus, if curcumin that is present in the PLGA micelle is aligned parallel to the hydrophobic part of CPB, the interaction between curcumin and the pyridinium ion will be favored. This is in agreement with what is reported earlier when curcumin is encapsulated in liposomes.^21,22^ Hence, when the electron transfer process occurs, curcumin leaks the hydrophobic pocket of PLGA into the aqueous phase, thus leading to the decrease in the intensity of curcumin.

Stern–Volmer plots in the presence of CPB and KI are depicted in Figure 5C,D, respectively. A high quenching rate constant of curcumin by CPB was found to be 0.00125 μM^–1^, and it is similar to the value obtained when curcumin is quenched by CPB in F108 polymers.^1^

Similarly, curcumin was quenched by KI, but the quenching rate constant was equal to 1.55608 × 10^–6^ μM^–1^, smaller than that of CPB. This interaction with iodide may result from the hydrogen bonding present between PLGA and curcumin. Therefore, curcumin encapsulated inside the PLGA micelle is greatly quenched by the hydrophobic quencher CPB compared with the iodide quencher. This also confirms that curcumin is positioned in the hydrophobic pocket of the micelle at the Stern layer (see Figure 5E).

## 4 Conclusion

For the first time, the self-assembly behavior of PLGA polymer was investigated. Curcumin was utilized as a fluorescence probe to determine the CMC and CMT of PLGA, which were found to be 0.33 g/L and 25 °C, respectively. Curcumin was also used to study the effect of adding NaCl salt to the CMC value. It was found that the increase in NaCl concentrations reduced the CMC by around twofold from 0.25 to 0.14 g/L as the NaCl concentration reaches 150 mM. This is due to the salting out effect. The effect of solvent on the aggregation behavior of the PLGA polymer was studied using chloroform, and it was found that the polymer aggregates at a similar concentration as in the acetone–water mixture. However, the main difference was in the aggregation behavior where reverse micelles are obtained in chloroform instead of normal micelles. Finally, in order to determine the position of curcumin in the PLGA micelles, the fluorescence quenching experiment was conducted using two quenchers, KI and CPB. Based on the obtained results, it was concluded that curcumin is located near the hydrophobic pocket of the Stern layer of the PLGA micelle.
